# Evaluation of a cardiopulmonary resuscitation curriculum in a low resource environment

**DOI:** 10.5116/ijme.563a.5d85

**Published:** 2015-11-07

**Authors:** Mary P. Chang, Camila B. Lyon, David Janiszewski, Deborah Aksamit, Francis Kateh, John Sampson

**Affiliations:** 1 Department of Emergency Medicine, Johns Hopkins University, Baltimore, Maryland, USA; 2 Department of Anesthesiology/Pediatric Anesthesiology, Vanderbilt University, Nashville, Tennessee, USA; 3 University of Baltimore, Baltimore, Maryland, USA; 4 Office of Emergency Training, Response, and Evaluation, Johns Hopkins University, Baltimore, Maryland, USA; 5 Ministry of Health, Monrovia, Liberia; 6 Department of Anesthesiology/Critical Care Medicine, Johns Hopkins University, Baltimore, Maryland, USA

**Keywords:** Medical education, CPR, Liberia, low-resource environment

## Abstract

**Objectives:**

To evaluate whether a 2-day International Liaison Committee on Resuscitation (ILCOR) Universal Algorithm-based curriculum taught in a tertiary care hospital in Liberia increases local health care provider knowledge and skill comfort level.

**Methods:**

A combined basic and advanced cardiopulmonary resuscitation (CPR) curriculum was developed for low-resource settings that included lectures and low-fidelity manikin-based simulations. In March 2014, the curriculum was taught to healthcare providers in a tertiary care hospital in Liberia. In a quality assurance review, participants were evaluated for knowledge and comfort levels with resuscitation before and after the workshop. They were also videotaped during simulation sessions and evaluated on standardized performance metrics.

**Results:**

Fifty-two hospital staff completed both pre- and post-curriculum surveys. The median score was 45% pre-curriculum and 82% post-curriculum (p<0.00001). The median provider comfort level score was 4 of 5 pre-curriculum and 5 of 5 post-curriculum (p<0.00001). During simulations, 93.2% of participants performed the pulse check within 10 seconds, and 97.7% performed defibrillation within 180 seconds.

**Conclusion:**

Clinician knowledge of and comfort level with CPR increased significantly after participating in our curriculum. A CPR curriculum based on lectures and low-fidelity manikin simulations may be an effective way to teach resuscitation in this low-resource setting.

## Introduction

Cardiovascular disease is a leading cause of mortality and a major risk factor for sudden cardiac death (SCD) worldwide.^1^^,^^2^ SCD is defined as a cardiac-induced, unexplained death within 1 hour of symptom onset.^3^ There are 300,000 SCDs in Europe and 400,000 SCDs in the United States annually.^4^ In Africa, the incidence of SCD is unknown, but several studies are addressing this issue. The Pan-African SCD registry was formed in 2014, and Cameroon has recently established the Douala-Sudden Unexplained Death survey.^4^^,^^5^

Causes of cardiac arrest include ischemic cardiovascular disease and arrhythmias.^6^ Factors that improve outcomes of patients who experience cardiac arrest include early cardiopulmonary resuscitation (CPR), effective chest compressions, and early defibrillation.^7^^,^^8^ The odds ratio for improved victim survival with bystander-only CPR is 2.5.^9^ In the United States, the number needed to treat to prevent death with rapid defibrillation for SCD is 2.5.^10^^,^^11^ Critical care delivery is compromised when medical providers are unable to deliver basic life support to cardiac arrest patients. Thus, survival after cardiac arrest depends on quality of education and an effective healthcare system.

There is a paucity of published data on adult advanced life support knowledge and skills in Africa. A Nigerian hospital evaluated 366 nurses and confirmed that CPR skills deteriorate rapidly 1 year after training.^12^ South Africa showed improvement in CPR education between 2000 and 2010 with an AHA-accredited BLS training site that has 40 trainees per year.^13^^,^^14^ Currently, Liberia is rebuilding their healthcare system and needs to increase healthcare provider capacity.^15^ Liberia has experienced a negative flux of native healthcare providers from both the civil wars and Ebola outbreak. In 2003, Liberia concluded a series of civil wars that spanned over 2 decades and destroyed 95% of its healthcare infrastructure.^16^ The number of trained physicians in Liberia dwindled from 400 to 20 for a population of 3 million.^17^ The 2014 Ebola epidemic has further reduced the number of healthcare providers in the country. As of January 7, 2015, 178 healthcare workers in Liberia had died from Ebola.^18^

Sustaining the healthcare system for Liberians is dependent on competent, trained providers. Performing basic and advanced life support is an essential part of a healthcare provider’s scope of practice. Our goal is for BLS at minimum to be an essential skill among Liberian healthcare providers. Our team was invited by a rural, tertiary care hospital in Liberia to educate healthcare providers on resuscitation practices. In this study, we evaluated whether local health care providers had an increase in knowledge and skill comfort level after participating in our educational resuscitation workshop designed for low-resource environments.

## Methods

### Setting

Liberia is a West African country with an estimated population of 4,092,310 in 2014.^19^ The pre-Ebola United Nations Human Development Report ranked Liberia low on the human development index, at 175 out of 187 countries; the United States is ranked 5^th^.^20^ In 2006, the life expectancy in Liberia was 45 years, whereas that in the United States was 78 years.^21^ Jackson F. Doe (JFD) Regional Referral Hospital is a tertiary care hospital near the border of Guinea and Cote d'Ivoire. The catchment area includes patients from the neighboring countries. Thus, the hospital is strategically located for the development of a sustainable medical education program with the potential to help an international and rural population that has limited access to health care. Since its opening in 2011, the hospital has been endowed with a number of technological resources, including four mechanical ventilators, a computerized tomography (CT) scanner, a fluoroscopy suite, and six manual defibrillators. The hospital did not establish training for defibrillator use, resuscitation teams, or a system for responding to a cardiac arrest. At the request of the hospital administration, a team of American Heart Association (AHA)-certified medical providers trained JFD hospital staff in basic and advanced life support skills, use of the defibrillators, and basic critical care.

### Participants

The curriculum was taught to 75 staff members, 52 of whom completed both the pre-curriculum and post-curriculum surveys and 3 did not fill out either survey. Table 1 shows the characteristics of the course participants. Participants had a median age of 34 years (range, 24–62 years), and most were female (58.3%). Occupations included technicians (14%), nurses (55%), and physicians (20%). Most participants had some form of prior CPR experience or education (55.6%).

**Table 1:** Characteristics of study participants (N=72)

Characteristic		Study Participants n (%)
Age (years, n = 67)		
	20–29	13 (19.4)
	30–39	38 (56.7)
	40–49	10 (14.9)
	50–59	5 (7.5)
	60–69	1 (1.5)
Sex		
	Male	30 (41.7)
	Female	42 (58.3)
Occupation		
	Physician	14 (19.7)
	Nurse	39 (54.9)
	Technician	10 (14.1)
	Midwife	3 (0.04)
	Physician assistant	2 (2.8)
	Pharmacist	1 (1.4)
	Anesthetist	2 (2.8)
Prior CPR Experience		
	Yes	35 (55.6)
	No	28 (44.4)

### Study design

In March 2014, a 2-day resuscitation curriculum based on the International Liaison Committee on Resuscitation(ILCOR) Universal Cardiac Arrest Algorithm^22^ was offered to all healthcare providers practicing at JFD hospital. Instructors were two anesthesiologists, an emergency medicine physician, and an emergency medicine nurse who each had at least 3 years of AHA certification. Students included physicians, nurses, and support staff (i.e., laboratory technicians). Two identical sessions were offered to accommodate the hospital staffing schedule.

Lectures covered basic life support (BLS) and advanced cardiac life support. Terminology was consistent with that of the international Utstein consensus conferences, which has published several recommendations on standardizing CPR educational material content, quality, documentation, and quality control.^8^ The only change was to teach assisted ventilation with a bag-mask device instead of mouth-to-mouth resuscitation. Liberia has three times the global average prevalence of tuberculosis.^23^

Students practiced chest compressions on low-fidelity manikins after the BLS lecture. The manikins produce a "click" when a chest compression of adequate depth is delivered. During the afternoons, they practiced megacode scenarios in groups to demonstrate resuscitation knowledge and skills. Simulation scenarios included ventricular fibrillation, respiratory arrest, and pulseless ventricular tachycardia.

### Data collection

Participants were surveyed on medical knowledge and comfort levels with skills before and after teaching the curriculum. The written survey contained questions that queried demographic factors, prior resuscitation training, level of medical training, current level of comfort delivering CPR to specific age groups, and knowledge regarding CPR skills. The questions on participants' comfort with performing CPR and knowledge to teach CPR were rated on a Likert scale from 1 to 5. Knowledge questions were multiple choice. Participants were given the same survey at the end of the 2-day workshop to assess improvement in comfort level and resuscitation knowledge.

For the skills component, participants were video recorded during simulation sessions for evaluation of quality metrics, including chest compression fraction (CCF), compression rate, time to chest compression, and time to defibrillation. An instructor present during the simulation provided real-time feedback. The video metrics were later recorded by one team member for analysis. The goal for the time from pulse check to chest compressions is 10 seconds and for pulse check to defibrillation is 3 minutes.^10^^,^^24^ The AHA recommends a CCF > 80% and a compression rate between 100 and 120 compressions per minute.^24^

### Data analysis

We used Microsoft Excel for data collection and STATA 12 (College Station, Texas) for statistical analysis. Statistical significance was set as p < 0.05**. **Descriptive statistics for demographics were calculated. Provider comfort level questions were based on a Likert scale of 1 to 5 and were calculated as ordinal data. We used ordered logistic regression to compare the post-curriculum provider comfort level score with the pre-curriculum score of the same individual. Knowledge scores were calculated as a percentage, and overall averages for pre-curriculum and post-curriculum were analyzed with the proportions test. We used logistic regression to evaluate the post-curriculum knowledge score with possible confounders such as the pre-curriculum score, sex, prior CPR education, age, and occupation. Age and pre-curriculum score were calculated as continuous variables. Sex and prior CPR education were classified as dichotomous variables. Occupations were converted to numbers (i.e., 1- physician, 2- nurse, 3- technician, 4- physician assistant, 5- midwife) and calculated as ordinal values. We used a paired t-test to determine if the change between each pre- and post-curriculum knowledge score was statistically significant. A Wilcoxon signed-rank test was used to evaluate comfort level scores pre- and post-intervention. For the video analysis, we used logistic regression to evaluate whether prior knowledge, prior CPR experience, post-training comfort level, age, occupation, or post-training test score had any effect on their ability to pass the competency. One team member reviewed the videos. Any hospital staff member who provided written consent was included in this research. The Johns Hopkins School of Medicine Institutional Review Board gave approval for this study.

## Results

### Evaluation of CPR knowledge

The aggregate median pre-curriculum score was 45%, and median post-curriculum score was 82%. A comparison of pre- and post-curriculum scores by paired t-test showed a 32% increase in scores (p<0.00001). Linear regression analysis of post-curriculum scores suggested that sex, prior CPR education (yes/no), age, and occupation had minimal effects on the post-curriculum scores; none were significant.

### Evaluation of CPR skills comfort level

The aggregate median provider comfort level score was 4 pre-curriculum and 5 post-curriculum. A Wilcoxon rank test showed that the increase in provider comfort level after the curriculum was statistically significant (p<0.00001). Ordered logistic regression analysis showed that prior CPR education, age, sex, occupation, and post-curriculum scores did not have a significant effect on CPR performance comfort level.

### Evaluation of CPR skills performance

Forty-four participants were video recorded during simulation sessions. Group size ranged from 3 to 8 participants. Three participants did not perform the pulse check. Forty-one participants performed the pulse check within 10 seconds. All but one participant defibrillated the manikin within 180 seconds. Logistic regression evaluation of the pulse-check-to-chest-compression time (n = 41) indicated that answering the question correctly on the post-curriculum survey suggested correct performance in a megacode scenario (p = 0.003). Prior CPR experience, post-curriculum comfort level, age, occupation, and post-curriculum test score did not affect whether the participant could perform pulse-check-to-chest-compression in less than 10 seconds (Table 2). The pulse-check-to-defibrillation time analysis (n = 40) did not show significant effect from factors such as prior CPR experience, post-curriculum comfort level, age, occupation, and post-curriculum test score (Table 3).

**Table 2:** Analysis of time from pulse check to chest compression (N=41)

Characteristic	Coefficient	*P* value
Compression test question	0.261	0.003
Prior CPR experience	-0.02	0.54
Comfort level	0.035	0.361
Post-curriculum test score	0.364	0.017
Age	0.001	0.617
Occupation	0.011	0.506

Forty-seven simulation sessions were recorded on videotape, with some participants participating in more than one session. CCF was >80% in five sessions (10.6%) and >70% in 33 sessions (70.2%). During simulations, 21 individuals (41.2%) performed chest compressions at 100–120 compressions/minute, 14 individuals (27.5%) were slower than 100 compressions/minute, and 16 individuals (31.4%) were faster than 120 compressions/minute. Logistic regression determined that providing adequate chest compressions per minute (100–120) was not statistically influenced by answering the question correctly on the post-course survey, prior CPR experience, occupation, sex, age, or post-course survey score (Table 4). However, the post-curriculum score coefficient was 1.9 (p = 0.084).

**Table 3:** Analysis of time from pulse check to defibrillation (N=40)

Characteristic	Coefficient	*P *value
Defibrillation test question	0.006	0.08
Prior CPR experience	0.002	0.954
Comfort level	-0.021	0.748
Post-curriculum test score	0.514	0.046
Age	-0.002	0.417
Occupation	0.014	0.635

**Table 4:** Analysis of chest compressions per minute (N=41)

Characteristic	Coefficient	P value
Occupation	0.05	0.756
Age	-0.003	0.829
Post-course comfort level	-0.075	0.760
Post-course survey score	1.90	0.084
Sex	-0.077	0.767
Prior CPR experience	0.209	0.388
Answer chest compression correctly on post-course survey	-0.352	0.411

## Discussion

Medical education in any setting should be evaluated for efficacy and quality per the Utstein Education in Resuscitation 2001 consensus recommendations. The international Utstein conferences are held to discuss uniform cardiac arrest reporting, but this symposium targeted improving the teaching of CPR.^25^ Recommendations from the symposium included educational material being targeted to specific populations, and that educational material quality control must be measured and re-evaluated. The resuscitation curriculum taught in Liberia included lectures and low-fidelity manikin simulation workshops, which have been shown to improve knowledge retention and performance in real code scenarios.^26^ The curriculum was designed for low-resource settings, emphasized equipment availability, and de-emphasized more advanced technologies, such as transvenous pacing. Its effectiveness was evaluated with pre- and post-course surveys that assessed knowledge about resuscitation and comfort level with resuscitation. Several factors, such as prior experience and age, can potentially influence curriculum evaluations.^27^

### Knowledge improvement

Our participants had a statistically significant increase in knowledge scores after participating in our resuscitation curriculum. The analysis of participants’ knowledge base indicated that variables such as age, sex, pre-curriculum score, prior CPR education, and occupation did not have a significant effect on post-curriculum score. Prior CPR experience did not improve a participant’s score on the post-curriculum survey, which was noted to be positive in a South African study.^14^ We did observe a statistically significant increase in CPR knowledge after the 2-day curriculum, leading us to believe that the overall improved scores on the post-course surveys were from knowledge gained during that workshop.

### Improved CPR skill comfort level

Comfort level of the participants was higher after the workshop. The participants ranked their skill comfort levels prior to the curriculum was high, but correlated poorly with knowledge scores. This could be that providers felt they could perform compressions, but did not have the knowledge base to lead a resuscitation effort. Learning or relearning the material is an important way for providers to feel comfortable with performing or teaching resuscitation. A systematic review of advanced life support knowledge and skill retention rates by Yang and colleagues suggested that both deteriorate between 6 and 12 months after training, with skills deteriorating faster than knowledge.^28^

### CPR Simulation session

The video analysis of resuscitation quality indicated that answering questions correctly on the post-tests could lead to better clinical performance. The participants’ ability to carry out defibrillation within 180 seconds of pulse check was not affected by prior CPR experience, post-curriculum comfort level, age, occupation, or post-curriculum test score. This finding suggests that practicing megacode simulations is as importance as knowing the guidelines.

The low rate of participants' ability to sustain CCF > 80% indicates that more practice is needed. The trainees practiced chest compressions on manikins individually before simulations, but the recorded sessions were their first experience working as a team. In future trainings, individuals should participate in several simulations to improve the time the patient receives chest compressions during cardiac arrest. More emphasis will be placed on “choreographing” the drills so that people performing chest compressions will switch only during pulse checks. Quality chest compressions are key to delivering effective CPR, and our results show that the 2-day workshop should dedicate more time to practicing simulations.

### Programmatic considerations

In a low-resource setting with a suboptimal number of medical providers, requesting staff to take time off is a difficult logistical task that requires advanced planning. Taking this into account, we would advocate restricting the workshop to 2 days, but scheduling more skills time.

During the curriculum implementation, super-users were selected and given one-on-one training and practice during the following week. The manikins were donated to the hospital so that super-users could teach new hospital staff on a regular basis, creating a sustainable education model.

Limitations Study limitations include a small sample size of 41 for skills analysis. Larger studies are needed to confirm our findings, although it could be difficult given the settings. Also, because of the Ebola outbreak shortly after the workshop, we were unable to complete the Utsein objective of reevaluating participants 6 months after the training course. However, during our visit, two participants demonstrated satisfactory advanced cardiac life support in a patient with real cardiac arrest. Analysis of cardiac arrest events in the hospital would be warranted in the future to measure educational program success. We plan to measure knowledge retention, refresh the knowledge of trainees, and build their resuscitation knowledge with other critical care education after the Ebola epidemic has resolved.

## Conclusions

After the 2-day CPR curriculum was presented at the JFD Regional Referral Center in Liberia, we measured a statistically significant increase in knowledge scores and participant comfort level with CPR. A CPR resuscitation curriculum based on lectures and low-fidelity manikin simulations may be an effective way to teach resuscitation in low-resource settings such as this. Additional research on education quality and retention rates of medical providers in low-resource settings is needed.

### Acknowledgements

Special thanks to Laerdal Medical (Wappingers Falls, New York, USA) for donating the simulation manikins. Our gratitude to Claire Levine who provided final edits to the manuscript.

### Conflict of Interest

The authors declare that they have no conflict of interest.
